# Immune Recovery Uveitis Masked as an Endogenous Endophthalmitis in a Patient with Active CMV Retinitis

**DOI:** 10.1155/2013/462968

**Published:** 2013-04-09

**Authors:** Ligia Figueiredo, Renata Rothwell, Miguel Bilhoto, Rosário Varandas, Sofia Fonseca

**Affiliations:** Department of Ophthalmology, Centro Hospitalar Vila Nova de Gaia/Espinho, Portugal

## Abstract

Cytomegalovirus (CMV) retinitis may occur in profoundly immunocompromised patients and be the initial AIDS-defining infection. The incidence and prevalence of CMV retinitis has declined substantially in the era of highly active antiretroviral therapy (HAART); nevertheless, it remains a leading cause of ocular morbility. We report the case of a 40-year-old man with blurred vision and pain in the right eye, three weeks after the initiation of effective HAART treatment. Ocular examination revealed a panuveitis causing an anterior chamber reaction with hypopyon and a dense vitreous haze. An endogenous endophthalmitis was suspected and treatment was ensued, without improvement. A vitreous tap was performed, and a positive polymerase chain reaction for CMV was found. A diagnosis of immune recovery uveitis (IRU) was made, and the patient responded to treatment with valganciclovir and dexamethasone. IRU is an intraocular inflammation that develops in patients with HAART-induced immune recovery and inactive CMV retinitis, although cases of active CMV retinitis have been described. Presentation with panuveitis and hypopion is rare and may be misleading regarding diagnosis and management.

## 1. Introduction

Cytomegalovirus (CMV) retinitis is a common opportunistic disease among patients with acquired immunodeficiency syndrome (AIDS) and typically manifests itself as progressive necrotizing retinitis with little or no intraocular inflammation [1–4]. Vitritis when present is usually mild and minimally symptomatic due to the severe immunodeficiency always associated with AIDS [1, 5].

 Due to the advent of highly active antiretroviral therapy (HAART), many patients may experience an immune reconstitution syndrome that can manifest itself as an ocular inflammatory response, termed immune recovery uveitis (IRU). This inflammatory response to CMV retinitis is a significant cause of visual morbidity in patients with AIDS [6–9].

We herein report a case of severe IRU with active CMV retinitis in a 40-year-old male infected with human immunodeficiency virus (HIV) and no known previous ophthalmological examination. When HAART was initiated, he developed signs of severe intraocular inflammation with panuveitis and hypopyon. 

## 2. Case-Report

A 40-year-old male presented with progressive visual loss, floaters, pain, and redness in the right eye for a week, with no other systemic complaints. He had been diagnosed with HIV-1 infection in 2010 and was being treated with abacavir, lamivudine, and efavirenz for six months (from March until August of 2012), but, due to low therapy adherence, he developed drug resistance. 

He then had a CD4^+^ T-lymphocyte count of 16 cells/*μ*L (normal 383–1347 cells/*μ*L) and an HIV viral load of 216000 copies/mL. 

A new HAART scheme with zidovudine, tenofovir, and atazanavir was implemented, together with cotrimoxazole and azithromycin prophylaxis. Ocular symptoms and complaints started 3 weeks after initiating the new HAART scheme. He had no known history of opportunistic infections.

**Figure 1:**
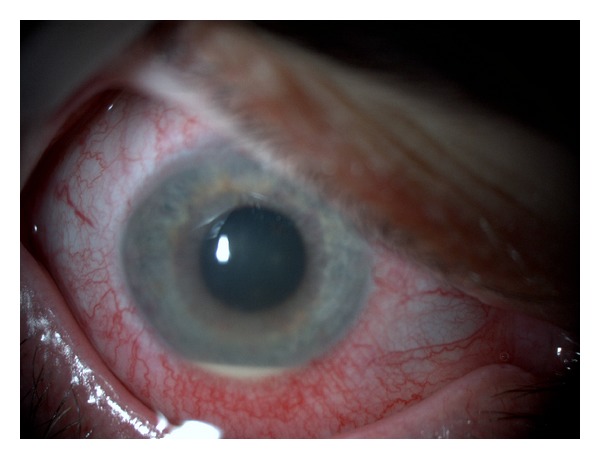
Initial presentation—anterior segment.

**Figure 2:**
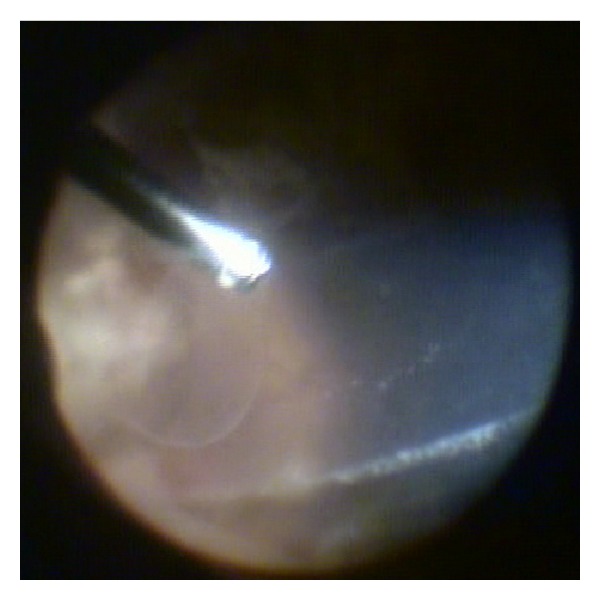
Picture taken during surgery showing severe vitritis.

On examination, his best corrected visual acuity was hand movements in the right eye (OD) and 10/10 in the left eye (OS). On slit-lamp examination, OS was normal and OD showed marked circumcilliary congestion, corneal edema, aqueous cell and flare grade 3, and a 2 mm mobile hypopyon. Intraocular pressure (IOP) with applanation tonometry was 8 mmHg OD and 14 mmHg OS. The ocular fundus examination of OD showed severe vitritis obscuring retinal details, and OS was normal. The systemic examination was unremarkable (Figures 1 and 2).

A clinical diagnosis of panuveitis secondary to endogenous bacterial or fungi endophthalmitis was suspected, and the patient started treatment with systemic ceftazidime, vancomycin and voriconazole, and intravitreal ceftazidime and vancomycin. After a negative gram and Giemsa staining, an aqueous humor tap showed no growth of bacteria or fungi. PCR virus analysis was not possible due to insufficient aqueous humor volume. Serology blood tests for syphilis were negative. Blood polymerase chain reaction (PCR) was negative for bacteria, fungi, and viruses. Hemocultures and urocultures were also negative. Blood count, erythrocyte sedimentation rate, interferon-*γ* release assay, and chest X-ray and CT were normal.

**Figure 3:**
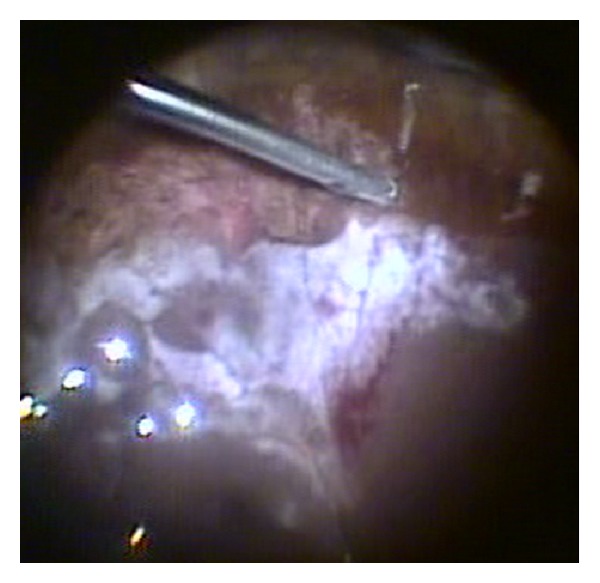
Picture taken during surgery showing signs of active retinitis.

After five days of treatment, there was no sign of improvement; so, the patient underwent a *pars plana* vitrectomy. The surgery consisted of a *pars plana* vitrectomy (as complete as possible) together with complete lensectomy (including removal of the capsular bag) and no intraocular lens (IOL) implant. During surgery, scattered, granular, yellow-white areas of retinal necrosis with patchy haemorrhages typical of active CMV retinitis were visible in the superior periphery. The superior peripheral retina broke due to severe necrosis originating a giant retinal tear which was treated with laser photocoagulation (Figure 3). Due to the retinal tear, an air fluid exchange was performed, and the intraocular antibiotics were slowly dropped into the eye, to avoid retinal detachment.

A vitreous tap performed during surgery showed no growth of bacteria or fungi. Vitreous PCR was positive for cytomegalovirus and negative for toxoplasma, herpes simples virus, herpes zoster virus, mycobacterium, and cryptococcus.

**Figure 4:**
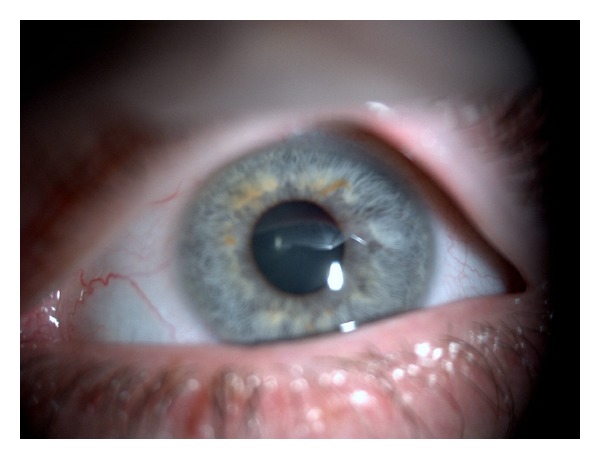
One month after treatment.

The patient was treated with oral valganciclovir and topical dexamethasone. The topical steroid was subsequently tapered over a period of six weeks, and the patient continued maintenance antiviral therapy with valganciclovir. On the last visit in December 2012, his best corrected visual acuity in OD was 4/10. The anterior segment inflammation and vitritis had subsided, and the CMV retinitis had clinically regressed, with no sign of retinal necrosis (Figure 4). An anterior chamber intraocular lens will be implanted after complete resolution of the intraocular inflammation.

## 3. Discussion

Before the HAART era, CMV retinitis developed in at least 30% of people with AIDS [10].

In the era of HAART, the incidence of CMV retinitis has declined by approximately 75% to 90% [11–13], but it remains the leading cause of ocular morbidity [14].

CMV retinitis may be the initial AIDS-defining opportunistic infection in 1.8%–3% of patients [15, 16]. CMV retinitis occurs only in profoundly immunocompromised HIV-infected patients, with CD4^+^ T-lymphocyte counts of less than 100 cells/mm^3^, but usually less than 50 cells/mm^3^[5, 16]. IRU is a noninfectious intraocular inflammation which develops in patients with CMV retinitis (or other intraocular infections, such as toxoplasmosis or tuberculosis) who have a substantial increase in CD4^+^ T-lymphocyte several weeks after starting HAART, even though it may develop months to years after an immune recovery with HAART. It is caused by a response to CMV antigens, which is made possible by the immune recovery. It usually develops in patients with inactive CMV retinitis; however, it can rarely occur in eyes with active CMV retinitis, particularly at the onset of inflammation, and such cases can be difficult to manage [16]. In a large clinical center cohort study that evaluated the prevalence and risk factors for IRU none of the 50 patients with CMV and IRU, showed signs of active retinitis [17].

An estimate of the incidence of IRU based on a large single-center study has varied from 0.11 per person-year to 0.83 per person-year [6, 7, 9]. In a 19-clinical-center cohort study of 259 patients with CMV retinitis, IRU occurred in 9, 6% of those who had immune recovery [17].

Known risk factors for IRU are larger lesions and previous use of cidofovir [17, 18].

The severity of inflammation in patients with IRU varies markedly and has been thought to be related to various factors including degree of immune constitution, extent of CMV retinitis, amount of intraocular CMV antigen, and previous treatment [19]. There may also be severe visual loss due to complications of inflammation such as macular edema, epiretinal membranes, neovascularization of the retina or optic disk, posterior synechiae, and cataract. Symptoms include floaters and/or visual loss [20].

In our case, the patient had an active CMV retinitis and severe ocular inflammation, indicating an immune reconstitution. This was evidenced by an increase in levels of CD4^+^ T-lymphocyte (from 16 cells/*μ*L to 101 cells/*μ*L in one month) and a decrease in viral load (from 216000 copies/mL to 650 copies/mL). The patient probably has achieved only limited CMV immunity: enough to mount an inflammatory response against CMV, but insufficient to prevent its reproduction in the retina.

Other ocular pathogens and mechanisms can cause similar intraocular inflammation such as endogenous endophthalmitis, the use of cidofovir, and IRU in patients with tuberculosis and toxoplasmosis. In our case, we attribute the hypopyon and posterior segment inflammatory response to increased immune function as a result of HAART because no other ocular pathogens besides CMV were found, the lesions in retina were suggestive of CMV retinitis, and the patient had not undergone treatment with cidofovir.

Initiation of HAART should be delayed until after the induction phase of anti-CMV therapy, as the reduction of antigen load with anti-CMV agents may reduce potential risk of IRU [16].

There are several anti-CMV drugs available including ganciclovir and its prodrug valganciclovir, foscarnet, cidofovir, and fomivirsen. The most commonly used are the ganciclovir intraocular implant and oral valganciclovir. The implant has the advantage of not causing systemic toxicity, but it does not protect against systemic CMV or contralateral CMV retinitis.

Treatment starts with high-dose induction therapy. Maintenance treatment should be continued until immune recovery is achieved because none of the anti-CMV drugs available eradicate ocular and systemic CMV antigens in the immunocompromised patient. 

Anti-CMV therapy is important during immune recovery because it has been proved to be protective against the development of IRU by reducing the amount of CMV antigens in the retina, although it is has not shown a favorable cost-effectiveness ratio where there are no signs of CMV retinitis [16].

CMV retinitis screening is recommended at 3-month intervals in patients with a CD4^+^ T-lymphocyte count inferior to 50 cells/*μ*L because 15% of patients with active CMV retinitis are asymptomatic.

Our patient did not undergo any ophthalmological examination before starting HAART. If so, he could have been diagnosed with active CMV retinitis and HAART would have been delayed until after the induction phase and he could have had a less severe inflammatory reaction.

## 4. Conclusion

Although the incidence of CMV retinitis has markedly decreased with the improved immune function that has resulted from the institution of HAART, it remains an important problem in the HAART era; new cases continue to be seen, not only among those for whom this therapy is not available, but also in patients who develop resistance to it.

The prevalence of IRU is substantial among eyes of patients with immune recovery, and it is an important cause of visual morbidity.

IRU usually develops in patients with inactive CMV retinitis; however, it can rarely occur in eyes with active CMV retinitis (as seen in our case).

Our case demonstrates the importance of ophthalmological screening in all patients before the initiation of antiretroviral therapy, reducing the probability of a prominent inflammatory response in the presence of an active infection.
